# Association between lifestyle modifications and improvement of early cardiac damage in children and adolescents with excess weight and/or high blood pressure

**DOI:** 10.1007/s00467-023-06034-5

**Published:** 2023-06-22

**Authors:** Simonetta Genovesi, Elena Tassistro, Marco Giussani, Laura Antolini, Giulia Lieti, Antonina Orlando, Massimo Montemerlo, Ilenia Patti, Gianfranco Parati

**Affiliations:** 1 School of Medicine and Surgery, Milano-Bicocca University, 20100 Milan, Italy; 2 Cardiology Unit, Istituto Auxologico Italiano, IRCCS, 20100 Milan, Italy; 3 Bicocca Center of Bioinformatics, Biostatistics and Bioimaging (B4 Center), School of Medicine and Surgery, Milano-Bicocca University, 20100 Milan, Italy

**Keywords:** Children, Hypertension, Obesity, Left ventricular hypertrophy, Left ventricular geometry, Lifestyle

## Abstract

**Background:**

It is not known whether, in children and adolescents with alterations in weight and/or blood pressure (BP), lifestyle modifications are associated with an improvement of early cardiac damage.

**Methods:**

In a pediatric population referred for excess weight, high BP, or both (*n* = 278, 10.6 (2.3) years), echocardiography was performed at enrollment and after 15 months of follow-up, during which participants received nonpharmacological treatment, based on correcting unhealthy lifestyles and improving dietary habits. Left ventricular mass was indexed for height (g/m^2.7^, LVMI), and an LVMI value higher than or equal to age- and gender-specific 95^th^ percentile was the criterion for defining left ventricular hypertrophy (LVH). Multiple linear and logistic regression analyses were carried out to determine associations between changes in BMI and BP z-scores and changes of LVMI values and LVH prevalence, from baseline to follow-up.

**Results:**

At baseline, 33.1% of study participants were hypertensive, 52.9% obese, and 36.3% had LVH. At follow-up, the prevalence of hypertension, obesity, and LVH was 18.7%, 30.2%, and 22.3%, respectively (*p* < 0.001 for all). A decrease in LVMI from 37.1 to 35.2 g/m^2.7^ (*p* < 0.001) was observed. Only delta BMI z-score positively related to an improvement of LVMI. Reductions of BMI (OR = 0.22, 95% CI 0.07–0.64) and diastolic BP (OR = 0.64, 95% CI 0.42–0.93) z-scores from baseline to follow-up and family history of hypertension (OR = 0.36, 95% CI 0.16–0.78) were associated with a lower prevalence of LVH.

**Conclusions:**

In a pediatric population at cardiovascular risk, changing incorrect lifestyle and dietary habits is associated with both reduction of BMI and BP values and regression of early cardiac damage.

**Graphical abstract:**

**Figure:**
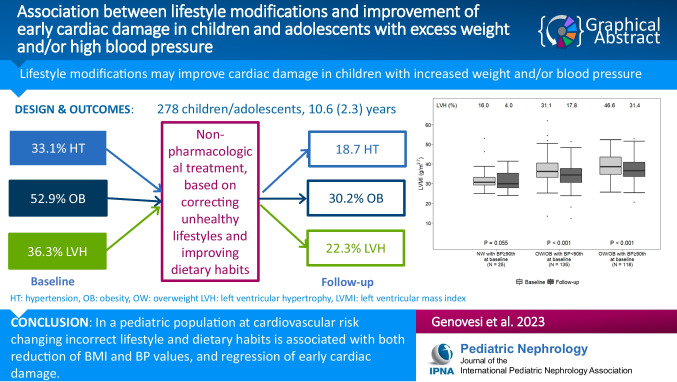
A higher resolution version of the Graphical abstract is available as Supplementary information

**Supplementary information:**

The online version contains supplementary material available at 10.1007/s00467-023-06034-5.

## Introduction

Evidence is available that left ventricular hypertrophy (LVH) is independently related to mortality and morbidity from cardiovascular diseases in adults and that arterial hypertension is the most important cause of LVH [1, 2]. In adult hypertensive patients with LVH, treatment-induced reductions of LV mass index are associated with lower rates of cardiovascular end points [3]. A tight control of blood pressure (BP) values is therefore fundamental to prevent the development of LVH and to promote its regression when already present [4].

In children and adolescents, indexed cardiac mass (LVMI) increases in a physiological manner as the subject grows. In this age group, LVH is the most frequent clinically detectable expression of organ damage in the presence of hypertension. Furthermore, excess weight is associated with increased LVMI and LVH even in the absence of hypertension [5–8]. In particular, it has been shown that obesity plays a greater role in determining LVH than hypertension [9]. European guidelines [10, 11] recommend starting drug therapy in children/adolescents with primary hypertension and organ damage. Some previous prospective studies have shown that the use of antihypertensive drugs leads to a reduction in the prevalence of LVH in hypertensive children [12–15], but there are no data regarding the efficacy of nonpharmacological treatment in this population. Interventions based on modification of incorrect lifestyles (i.e., bad eating habits, low level of physical activity, and excessive time spent in sedentary activities) may reduce both BP and body mass index (BMI) values in a pediatric population with excess weight and/or high BP. It should be noted that this approach was associated with improved BP in hypertensive-only children, decreased BMI in excess weight-only children, and improved BP and BMI values in children with both pathological conditions [15–17]. Studies using noninvasive indices of vascular health have shown that diet- and exercise-based interventions are able to lead to positive outcomes, in terms of reduced carotid intima-media thickness, improved endothelial function, and vascular stiffness [18, 19]. However, these are studies performed on small samples, and more extensive and definitive evidence is lacking on this topic. To our knowledge, no studies are available that evaluate the efficacy of nonpharmacological treatment in reducing cardiac organ damage in terms of both LVMI and the presence of LVH. The dietary-behavioral approach is the only tool that can be used in obese nonhypertensive children with LVH and represents a key intervention strategy measure in those with primary hypertension, particularly when associated with excess weight. Therefore, it is of particular interest to understand whether this approach is effective in children with hypertension and/or excess weight presenting with LVH.

The present study was carried with the aim to fill this gap and to assess whether nonpharmacological treatment in children and adolescents presenting with hypertension, excess weight, or both risk factors together is associated with a reduction of LVMI and the prevalence of LVH. We sought to assess whether this approach is related to improved cardiac parameters both in children with only one disease condition (elevated blood pressure or excess weight) and in those with both conditions (elevated blood pressure with excess weight). The association between nonpharmacological treatment and LV geometry changes is also investigated.

## Materials and methods

### Subjects

The study population consisted of a cohort of children and adolescents aged 4–18 years, who, because of the presence of high BP values and/or excess weight, had been consecutively referred to the Unit for Cardiovascular Risk Assessment in Children from 16/12/2012 to 12/09/2017. Children were referred by family pediatricians who follow children of all sociodemographic conditions free of charge. This Unit is public and access is free, as is the care provided. The following exclusion criteria did not allow enrollment: diabetes, impaired glucose tolerance, secondary hypertension of any kind, and ongoing antihypertensive drug treatment. Children and adolescents were included in our study after their condition of excess of weight and/or BP elevation (systolic (SBP) and/or diastolic (DBP) ≥ 90^th^ percentile) had been confirmed both during the repeated visits performed by their family pediatrician and during two further clinic assessments performed in our center, at the initial visit and at the time of cardiovascular examination (baseline). Children with normal BP and normal weight at the recruitment visit were not included in the study. Children for whom it was necessary to start drug treatment to control BP values during follow-up were excluded from the final analyses. The Unit for Cardiovascular Risk Assessment in Children consists of the following members: a pediatrician, a nephrologist, a cardiologist, and a nutrition expert.

### Baseline and follow-up assessments

In all children, the following parameters were examined at baseline and at the end of the 15-month follow-up period: weight, height, waist circumference (WC), and BP. In addition, a cardiac echocardiographic examination was performed at the same time points. Between the baseline and the final assessment, a minimum of three and a maximum of six additional periodic visits (about one every three months) were performed, during which the same variables were recorded again. At each, visit information was requested from family members in order to evaluate compliance with the modifications requested by the study: change in diet, increment in physical activity, and reduction in time spent watching television or playing videogames, and the answers were recorded in the participants’ medical record. If deemed necessary, reinforcement of dietary and of lifestyle recommendations was given. In case there were any doubts or problems outside the time of visits, parents had the possibility to contact clinical team members (physicians or nutritionists) via e-mail and obtain the necessary explanations.

### Anthropometric parameters and blood pressure

The measured anthropometric parameters were height, body weight, and WC. Applied precision for data recording was 100 g for body weight and 1 cm for height. Body mass index was then calculated as the weight in kilograms divided by the square of height in meters. Body mass index z-scores were calculated with the Centers for Disease and Control prevention charts (https://www.cdc.gov/growthcharts/clinical_charts.htm; last accessed January 9, 2023). Weight class was defined according to the classification by the International Obesity Task Force [20] distinguishing between normal weight (NW), overweight (OW), and obese (OB) individuals. Waist circumference was recorded with a precision of 1 cm while the study participant was standing. The waist measurement was done as recommended by Lohman et al. [21]. Waist-to-height ratio (WtHr) was obtained by dividing WC by height. Blood pressure was measured with a specific oscillometric device validated for use in children (Omron 705IT; Omron Co, Kyoto, Japan); particular care was taken to use the appropriate cuff size.

Blood pressure values were recorded after a resting period of at least 5 min and while the participants were sitting. The measurement was carried out 3 times (at intervals of a few minutes), and the mean value of the second and third measurements was recorded. Percentiles and z-scores of SBP and DBP were calculated on the basis of the nomograms of the National High Blood Pressure Education Program (NHBPEP) Working Group on High Blood Pressure in Children and Adolescents [22]. The children were classified according to the mean of the two measurements as follows: normotensive (NT) if both SBP and DBP percentiles were < 90^th^; high-normal (HN) if SBP and/or DBP percentiles were ≥ 90^th^, but both < 95^th^; hypertensive (HT) if SBP and/or DBP percentiles were ≥ 95^th^.

The study population was divided into three subgroups based on BP category and weight class at baseline, in the following manner: NW children with normal weight but with BP values ≥ 90^th^ percentile = HN + HT (elevated BP only); OW/OB children with excess weight but with BP values < 90^th^ percentile = OW + OB (excess weight only); OW/OB children with both excess weight and with BP values ≥ 90^th^ percentile = OW/OB + HN/HT (elevated BP + excess weight).

### Echocardiography

Two-dimensional M-mode echocardiographic evaluations were performed in the standard precordial positions using digital echocardiography equipment according to the standard M-mode measurement recommendations [23]. Instantaneous measurements were made over three cardiac cycles, and the average values were obtained from each subject for left atrial end-diastolic diameter (LAd), interventricular septum thickness at end-diastole (IVSd), left ventricular posterior wall thickness at end-diastole (LVPWd), and left ventricular end-diastolic diameter (LVEDd). Left ventricular mass was calculated according to the convention of the American Society of Echocardiography and subsequently indexed (LVMI) to height (m^2.7^) [24].

Left ventricular hypertrophy was diagnosed in the presence of an LVMI greater than or equal to the age- and gender-specific 95^th^ percentile [25]. Relative wall thickness (RWT) was calculated using the formula RWT = (IVSd + LVPWd/LVEd). This parameter was then adjusted for age, according to the formula RWT (adj) = RWT − 0.005 * (age − 10), specific for subjects with age 1–17 years [26].

Left ventricular geometry was evaluated on the basis of values of LVMI and RWT (adj): the threshold for increased LVMI was the age- and gender-specific 95^th^ percentile [25]; the threshold for increased RWT (adj) was the age-specific 95^th^ percentile, 0.375 [26]. Geometry was classified as normal (NG) in the presence of normal LVMI and normal RWT (adj), concentric remodeling (CR) in the case of normal LVMI and high RWT (adj), concentric hypertrophy (CH) when both LVMI RWT (adj) were increased, and eccentric hypertrophy (EH) in case of high LVMI and normal RWT (adj).

### Recommended lifestyle modifications

All participants were invited to engage in at least two to three hours of structured physical activity per week [27], to perform more unstructured spontaneous physical activity, and to diminish and shorten sedentary activities such as playing videogames or watching TV to a maximum of one hour per day, as recommended by the Italian Society of Pediatrics (https://sip.it/2017/09/25/non-solo-sport-ma-anche-gioco-la-piramide-dellattivita-fisica-e-motoria-per-combattere-lobesita/). Children were encouraged to choose sports disciplines of their liking for an appropriate number of hours per week.

General advice was given to all participants on how to obtain a healthy and balanced diet (more fruit and vegetables, low-fat dairy products, less intake of free sugars, and elimination of soft drinks) with a correct salt intake (a maximum of 5 g/day which equals about 2 g of sodium) following the World Health Organization (WHO) guidelines (www.who.int/nutrition/publications/guidelines/sodium_intake/en/).

At the baseline visit, the parents of the children and adolescents were interviewed by an expert nutritionist in order to evaluate the eating habits and the degree of physical activity of the participants. On the basis of the information that was obtained, appropriate and personalized changes in lifestyle and nutritional habits were proposed to each participant. Once it was determined what the appropriate caloric intake was and how it was to be divided as protein, glycides, and lipid intake, specific discussions between the child, parents, and nutritionist were carried out to be able to arrive at a diet that took into account the preferences of the individual child and the needs of families. A personalized dietary scheme was prepared for all participants with the help of a specifically designed software (Dietosystem, DS Medica, Milano).

Depending on the actual body weight and BP status of each participant (isolated excess weight, or elevated, or a combination of both risk factors), specific additional recommendations were given.

#### Excess weight

Overweight and OB individuals received a weekly dietary program, the caloric content of which had been previously calculated by the Schofield equation [28] which considers the subjects’ basal metabolic rate and according to functional metabolism [29]. Young children were invited to follow a balanced normocaloric regimen that would equal to the estimated energy expenditure, while severe overweight adolescents were recommended to follow a mildly hypocaloric diet (− 10%).

#### Elevated blood pressure

In HN and HT individuals, it was proposed to reduce salt intake to less than 5 g per day, following the WHO guidelines.

The study protocol was approved by the Local Ethics Committee (Istituto Auxologico, Milano) (RICARPE 2015_10_20_02), and informed consent was obtained from the children’s parents.

### Statistical analysis

The characteristics of study participants are reported as means and standard deviation (SD) or as median and interquartile range (IQR) in the case of continuous variables and as frequencies and percentages for categorical variables. Univariate analyses to compare the study variables at baseline and at follow-up were conducted through t-tests for paired data or Wilcoxon signed-rank tests for continuous variables and by the McNemar test for categorical variables.

The distributions of weight class and BP category at baseline and at follow-up were described by bar plots, and the proportions were compared by an extension of the McNemar test for the marginal homogeneity of categorical data with more than 2 levels [29]. The distribution of the difference between BMI at follow-up and BMI at baseline according to weight classes at baseline was represented through boxplots and the proportions of OW, and OB children at baseline with a BMI reduction were compared with the chi-square test. The distribution of the difference between SBP at follow-up and SBP at baseline according to BP categories at baseline was represented through boxplots, and the proportions of HN and HT children at baseline with an SBP reduction were compared with the chi-square test. The distribution of LVMI was represented at baseline and at follow-up by boxplots separately on the basis of the weight and BP status defined at baseline. Median values were compared through the Wilcoxon signed-rank test. In addition, the percentage of LVH in each group of patients was displayed.

Multiple linear regression models were used to assess the impact of the relevant factors on the difference of LVMI from baseline to follow-up. The same models were implemented also separately on patients without and with LVH.

Multiple logistic regression models were used to assess the impact of the relevant factors on the prevalence of LVH at follow-up.

A multiple multinomial regression model was used to estimate the influence of LV geometry at baseline, the difference between BMI z-score at baseline and at follow-up, and the difference between SBP (or DBP) z-score at baseline and at follow-up on LV geometry at the end of the study [30]. Plots of the predicted probabilities of being in each category of cardiac geometry at follow-up, stratified by geometry category at baseline, varying the values of BMI or SBP/DBP Δz-scores (keeping fixed SBP/DBP Δz-score = 0 or BMI Δz-score = 0, respectively) are displayed.

Statistical analyses were performed with R 4.0.3 (http://www.R-project.org). All *p* values were 2-sided, with *p* values < 0.05 considered statistically significant.

## Results

A total of 304 children performed the baseline assessment. Twenty-six children were subsequently excluded from the analysis even if they completed the follow-up assessment; 12 of them needed to start drug treatment, while in 14 echocardiographic evaluation could not be obtained either at baseline or at follow-up.

**Fig. 1:**
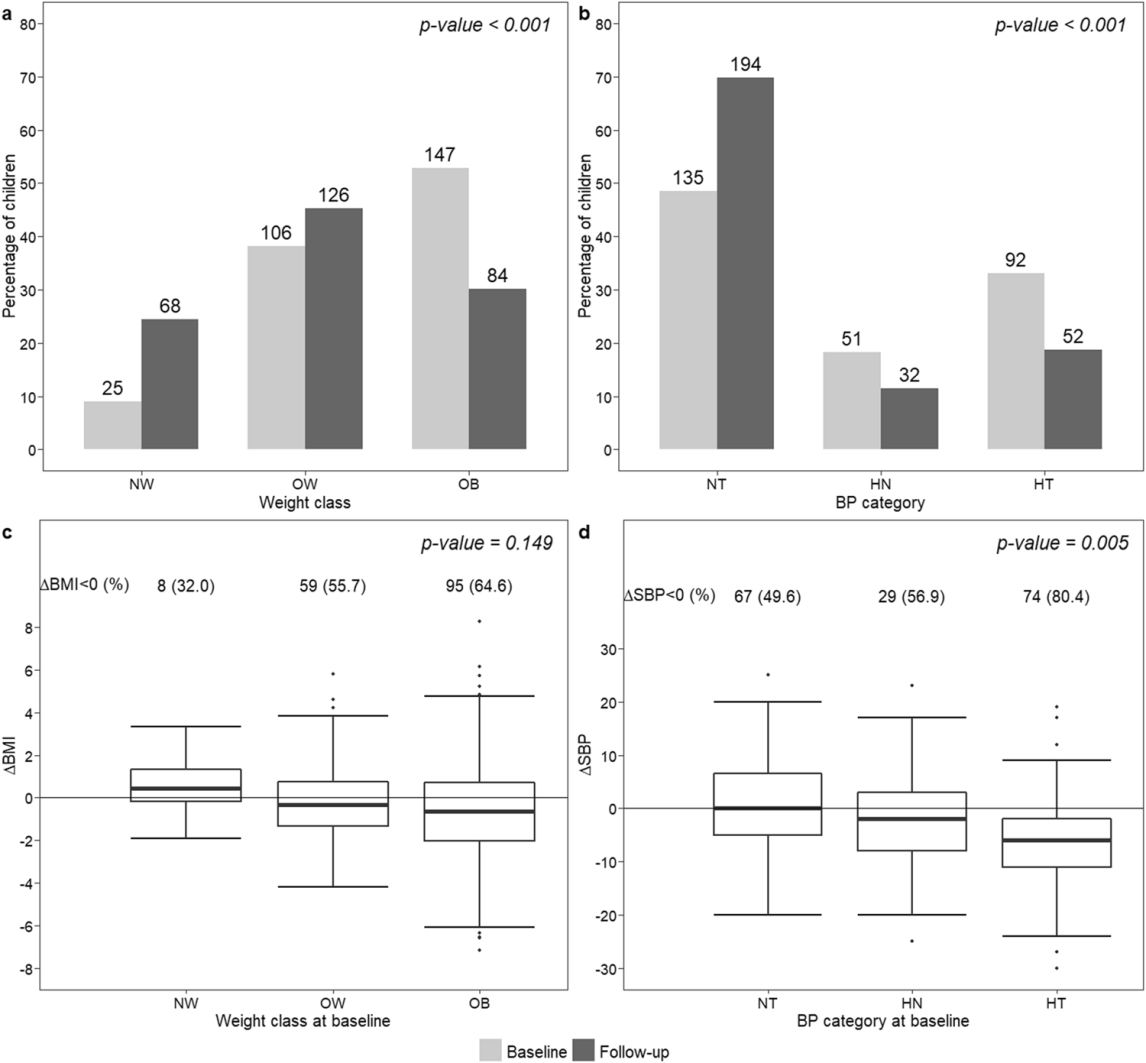
**a** Distribution of weight classes at baseline and at follow-up. **b** Distribution of blood pressure categories at the same time points as **a**. **c** Distribution of the change in BMI between baseline and follow-up according to weight classes at baseline. **d** Distribution of the change in SBP between baseline and follow-up according to BP categories at baseline

A diagram describing the process of patient selection of the study participants and their follow-up is shown in Figure S1. The study sample thus consisted of 278 children and adolescents with a mean age of 10.6 (SD = 2.3) years. Slightly more than half of the study population were male and 56% were prepubertal (Table 1). At baseline, 9.0% of the population was NW (*n* = 25), 38.1% was OW (*n* = 106), and 52.9% (*n* = 147) was OB (Fig. 1a). In 73.1%, WtHr was > 50%. Children with normal BP values were 48.6% (*n* = 135), 18.3% were HN (*n* = 51), and 33.1% (*n* = 92) were HT (Fig. 1b). Of the 92 HT children, 74 (51.7%) showed systolic HT, 5 (3.5%) diastolic HT, and 13 (9.1%) were both systolic and diastolic hypertensive. Thirty-five percent of the subjects had at least one hypertensive parent. After a median follow-up period of 14.7 (12.4, 19.3) months, during which the children received nonpharmacological treatment and were followed up with periodic quarterly visits, the z-scores of BMI, SBP, and DBP were significantly reduced (*p* < 0.001). The percentage of children who had started pubertal development increased to 73.3% (Table 1).

**Table 1:** Anthropometric and clinical characteristics and echocardiography parameters at baseline and follow-up

Parameter	Baseline (*N* = 278)	Follow-up (*N* = 278)	*p*
*Anthropometric and clinical characteristics*			
Age (years), mean (SD)	10.6 (2.3)	12.1 (2.4)	< 0.001
Gender (males), *n* (%)	150 (54.0)	–	
Puberty yes, *n* (%)	120 (44.0)	178 (73.3)	< 0.001
Weight (kg), median (IQR)	51.8 (41.3, 62.6)	57.4 (45.7, 68.5)	< 0.001
Height (cm), mean (SD)	145.6 (14.4)	153.5 (13.6)	< 0.001
BMI, mean (SD)	24.6 (4.3)	24.2 (4.5)	0.006
BMI (z-score), median (IQR)	1.9 (1.4, 2.1)	1.5 (1.0, 1.9)	< 0.001
Waist (cm), mean (SD)	78.7 (11.8)	78.3 (11.7)	0.106
WtHr (%), mean (SD)	54.1 (6.6)	51.0 (6.6)	< 0.001
Systolic BP (mmHg), mean (SD)	117.4 (12.6)	115.4 (12.2)	< 0.001
Diastolic BP (mmHg), mean (SD)	68.2 (8.4)	66.8 (8.0)	0.014
Systolic BP (z-score), mean (SD)	1.2 (1.0)	0.8 (1.0)	< 0.001
Diastolic BP (z-score), mean (SD)	0.6 (0.7)	0.3 (0.7)	< 0.001
Family history of hypertension, *n* (%)	96 (34.8)	–	
Follow-up time (months), median (IQR)	15.0 (12.5, 20.6)	–	
*Echocardiography parameters*			
IVS, median (IQR)	0.80 (0.70, 0.88)	0.81 (0.70, 0.90)	0.003
PWT, median (IQR)	0.76 (0.70, 0.80)	0.80 (0.70, 0.80)	0.004
LVEDD, median (IQR)	4.30 (4.00, 4.50)	4.40 (4.20, 4.70)	< 0.001
LVMI (g/m^2.7^), median (IQR)	37.06 (33.03, 41.62)	35.19 (30.93, 38.72)	< 0.001
LVH, *n* (%)	101 (36.3)	62 (22.3)	< 0.001
Adjusted RWT, mean (SD)	0.36 (0.32, 0.39)	0.35 (0.32, 0.38)	< 0.001

*BMI* body mass index, *BP* blood pressure, *IQR* interquartile range, *IVS* interventricular septum, *LVEDD* left ventricular end-diastole diameter, *LVH* left ventricular hypertrophy, *LVMI* left ventricular mass index, *PWT* posterior wall thickness, *RWT* relative wall thickness, *SD* standard deviation, *WtHr* waist-to-height ratio

The distribution of weight classes at the end of follow-up was NW 24.5%, OW 45.3%, and OB 30.2%, which was significantly different from the percentages observed at baseline (*p* < 0.001, Fig. 1a). The percentage of children with WtHr > 50% at follow-up was reduced to 52.0% (*p* < 0.001). Individuals who had BP values in the normal range at follow-up were 69.8%, those with HN were 11.5%, and those with HT accounted for 18.7% of the sample. All percentages were significantly different from the corresponding values at baseline (*p* < 0.001, Fig. 1b). We observed an increment (absolute value) of the delta BMI z-score moving from NW to OW and OB (Fig. 1c, *p* = 0.149) and of delta SBP z-score moving from NT to HN and HT (Fig. 1d, *p* < 0.005).

Table S1 shows the change in the distribution of weight and BP subgroups in the study population from baseline to follow-up. It is interesting to note that at the end of the follow-up period, 17.6% of children did not show abnormal values, either for weight or for BP, whereas the proportion of subjects with BP values ≥ 90th percentile and excess weight decreased from 42.4% to 23.4%.

**Fig. 2:**
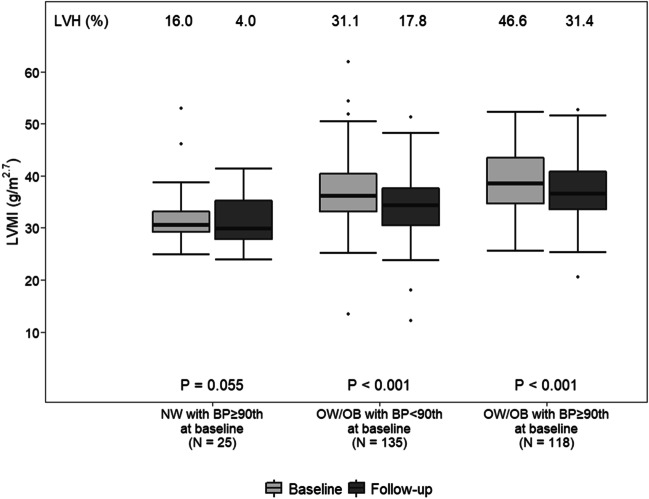
Distribution of left ventricular mass index values and prevalence of left ventricular hypertrophy at baseline and at follow-up according to weight and blood pressure subgroups defined at baseline. *BP*, blood pressure; *LVH*, left ventricular hypertrophy; *LVMI*, left ventricular mass index

Table 1 shows the echocardiographic parameter values of the study population at baseline and at follow-up. Despite the expected increase in raw values of wall thickness due to growth, both LVMI and adjusted RWT were significantly lower at the end of the follow-up period (*p* < 0.001). The percentage of children with LVH decreased by 14% (*p* < 0.001). Baseline and follow-up values of LVMI and the percentage of LVH according to the baseline clinical subgroups are shown in Fig. 2. At baseline, the lowest prevalence of LVH was observed in children with BP values ≥ 90th percentile (16.0%; elevated BP only); the prevalence was higher in those OW or OB (31.1%; excess weight only) and highest in the group of individuals with BP values ≥ 90th percentile and excess weight (46.6%; elevated BP and excess weight; *p* < 0.001 between groups). The prevalence of LVH at the end of the follow-up period in children with elevated BP only, weight excess only, or both risk factors together at baseline was 4.0%, 17.8%, and 31.4%, respectively, with the *p* values for the difference from the corresponding percentages at baseline being *p* = 0.055, *p* < 0.001, and *p* < 0.001, respectively. Thirty-eight children had concurrent HT (SBP and/or DBP ≥ 95th percentile) and LVH at baseline, two in the subgroup with elevated BP only and 36 in the subgroup with elevated BP + excess weight. At the end of follow-up, 20 children out of 38 already had LVH, but only 11 of them still had BP values ≥ 95th percentile.

In Table 2, results from multivariate linear regression analysis of the change in LVMI from baseline to the end of the study are shown, both of the whole sample and separately in children without or with LVH at baseline. In the overall population, delta BMI z-score was the only factor significantly associated with reduced LVMI (− 4.084 g/m^2.7^ per point decrease in z-score). Even in the subsample of children without LVH, the reduction in BMI z-score was the only independent predictor of LVMI reduction at follow-up (− 3.974 g/m^2.7^ per point decrease in z-score). In subjects with LVH at baseline, besides BMI z-score reduction (− 3.958 g/m^2.7^ per point decrease in z-score), family history of hypertension was also significantly related with reduced LVMI values at the end of follow-up (*p* < 0.004). Finally, for a one-year increase in the age, follow-up LVMI values increased by 0.417 g/m^2.7^ (*p* = 0.054). When the follow-up duration or the transition to prepubescent to pubescent was entered as an adjustment to the statistical models, the results were completely overlapping (Tables S2 and S3).

**Table 2:** Effect of gender, age, family history of hypertension, BMI, systolic BP (Model A), or diastolic BP (Model B) on the change in left ventricular mass index between baseline and follow-up in all subjects and separately in children without and with left ventricular hypertrophy at baseline, assessed by multiple linear regression analysis

Variable	Model A			Model B		
	*b*	(95% CI)	*p*	*b*	(95% CI)	*p*
*All subjects*						
Intercept	− 1.647	(− 4.372; 1.078)	0.235	− 1.643	(− 4.373; 1.087)	0.237
Gender (males)	0.359	(− 0.739; 1.457)	0.520	0.356	(− 0.743; 1.455)	0.524
Age (years)	0.072	(− 0.170; 0.314)	0.558	0.082	(− 0.158; 0.323)	0.501
Family history of hypertension	− 0.723	(− 1.874; 0.428)	0.217	− 0.727	(− 1.891; 0.417)	0.210
BMI (Δz-score)	− 4.084	(− 5.655; –2.514)	< 0.001	− 4.008	(− 5.574; − 2.442)	< 0.001
Systolic BP (Δz-score)	0.221	(− 0.385; 0.826)	0.474	–	–	–
Diastolic BP (Δz-score)	–	–	–	− 0.127	(− 0.776; 0.523)	0.701
*Children without LVH at baseline*						
Intercept	2.034	(− 1.059; 5.126)	0.196	2.040	(− 1.049; 5.130)	0.194
Gender (males)	0.258	(− 1.014; 1.529)	0.690	0.258	(− 1.017; 1.533)	0.690
Age (years)	− 0.181	(− 0.454; 0.092)	0.192	− 0.183	(− 0.454; 0.088)	0.184
Family history of hypertension	0.338	(− 0.991; 1.667)	0.616	0.335	(− 0.993; 1.664)	0.619
BMI (Δz-score)	− 3.974	(− 5.713; − 2.235)	< 0.001	− 3.983	(− 5.712; − 2.254)	< 0.001
Systolic BP (Δz-score)	− 0.041	(− 0.854; 0.773)	0.922	–	–	–
Diastolic BP (Δz-score)	–	–	–	0.001	(− 0.830; 0.833)	0.998
*Children with LVH at baseline*						
Intercept	− 6.933	(− 11.579; − 2.287)	0.004	− 6.800	(− 11.507; − 2.092)	0.005
Gender (males)	0.754	(− 1.039; 2.547)	0.406	0.781	(− 1.021; 2.583)	0.392
Age (years)	0.417	(− 0.007; 0.840)	0.054	0.422	(− 0.005; 0.848)	0.052
Family history of hypertension	− 2.676	(− 4.481; − 0.871)	0.004	− 2.762	(− 4.581; − 0.942)	0.003
BMI (Δz-score)	− 3.958	(− 6.980; − 1.223)	0.006	− 3.958	(− 6.843; − 1.074)	0.008
Systolic BP (Δz-score)	− 0.194	(− 0.394; 1.166)	0.328	–	–	–
Diastolic BP (Δz-score)	–	–	–	− 0.194	(− 1.085; 0.696)	0.666

Δz-score indicates the difference between the baseline value and the follow-up value

*BMI* body mass index, *BP* blood pressure, *CI* confidence interval, *LVH* left ventricular hypertrophy

^*^b indicates the multivariate coefficient

Logistic regression analysis performed on the whole sample identified the following independent predictors of lower LVH risk at follow-up: family history of hypertension (OR = 0.39 95% CI 0.17–0.83, Table 3, Model A and OR = 0.36 95% CI 0.16–0.77, Model B, *p* < 0.02), delta BMI z-score (OR = 0.22 95% CI 0.07–0.63, Table 3, Model A and OR = 0.22 95% CI 0.07–0.64, Model B, *p* < 0.01), and delta DBP z-score (OR = 0.63 95% CI 0.42–0.93, Table 3, Model B, *p* = 0.023). The presence of LVH at baseline was strongly and positively related with the probability of having LVH at follow-up (OR = 16.59 95% CI 8.02–37.22, Table 3, Model A and OR = 17.46 95% CI 8.35–39.76, Model B, *p* < 0.001).

**Table 3:** Effect of left ventricular hypertrophy at baseline, gender, age, family history of hypertension, BMI, systolic BP (Model A), or diastolic BP (Model B) on left ventricular hypertrophy at the follow-up by a multiple logistic regression analysis

Variable	Model A			Model B		
	OR	(95% CI)	*p*	OR	(95% CI)	*p*
LVH at baseline	16.586	(8.023; 37.220)	< 0.001	17.460	(8.353; 39.765)	< 0.001
Gender (males)	1.658	(0.825; 3.407)	0.160	1.683	(0.830; 3.492)	0.153
Age (years)	0.932	(0.791; 1.096)	0.395	0.915	(0.775; 1.078)	0.292
Family history of hypertension	0.389	(0.174; 0.827)	0.017	0.355	(0.155; 0.767)	0.011
BMI (Δz-score)	0.218	(0.070; 0.635)	0.006	0.218	(0.070; 0.639)	0.007
Systolic BP (Δz-score)	0.798	(0.558; 1.121)	0.201	–	–	–
Diastolic BP (Δz-score)	–	–	–	0.635	(0.424; 0.932)	0.023

Δz-score indicates the difference between the baseline value and the follow-up value

*BMI* body mass index, *BP* blood pressure, *CI* confidence interval, *LVH* left ventricular hypertrophy, *OR* odds ratio

Results were similar if visceral adiposity, WtHr, was entered into the model rather than BMI z-score (Table S4). An interaction term between BMI and BP was tested in all the models, but it resulted not statistically significant (data not shown). When the follow-up duration or the transition to prepubescent to pubescent was entered as an adjustment to the statistical models, the results were completely overlapping (Table S5–S8).

**Fig. 3:**
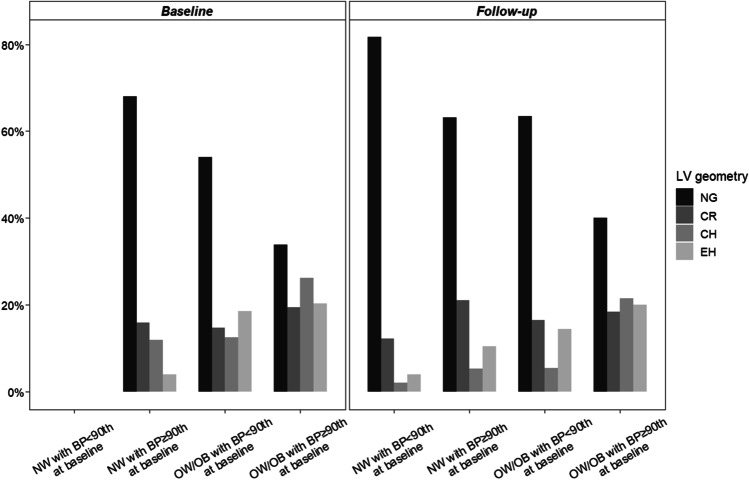
Distribution of left ventricular geometries in weight and blood pressure subgroups at baseline and at follow-up. *BP*, blood pressure; *CH*, concentric hypertrophy; *CR*, concentric remodeling; *EH*, eccentric hypertrophy; *NG*, normal geometry; *LV*, left ventricular

At baseline, 53.2% of the children had some alteration in cardiac geometry, whereas at study end the study participants with altered left ventricular geometry were 38.8%. Overall, cardiac geometric patterns improved from baseline to follow-up: NG from 46.8% to 61.2%, CR from 16.9 to 16.5%, CH from 18.3% to 8.6%, and EH from 18.0% to 13.7% (Table S9). The improvement in cardiac geometry was also evident within the different subgroups of weight and BP (Fig. 3).

The log odds of having CR (relative to NG) at follow-up for children with CR at baseline was predicted to be 2.321 points greater than that for those with NG at baseline. This means that subjects with CR at baseline were at greater risk of having CR also at follow-up and lower probability of having NG, as compared to those with NG at baseline (Table 4, Model 1). The log odds of having CH + EH (compared to NG) at follow-up for children with CR geometry at baseline was predicted to be 1.860 points greater than that for those with NG at baseline. The log odds of having CH + EH geometry (compared to NG) at follow-up for children with CH + EH geometry at baseline was predicted to be 3.188 points greater than that for those with NG at baseline (Table 4, Model 2). A one-unit decrease in the BMI z-score was linked to a decrease of 1.391 in the log odds of having CH + EH vs. NG at follow-up.

**Table 4:** Effect of left ventricular geometry at baseline, BMI, and systolic or diastolic BP on left ventricular geometry at the follow-up by a multiple multinomial regression analysis

Variable	*b*	RRR	(95% CI)	*p*	*b*	RRR	(95% CI)	*p*
	Model 1: CR vs. NG at the follow-up				Model 2: CH + EH vs. NG at the follow-up			
Intercept	− 1.844	0.158	(0.083; 0.301)	< 0.001	− 2.468	0.085	(0.036; 0.199)	< 0.001
LV geometry at baseline								
*CR vs. NG*	2.321	10.183	(4.413; 23.498)	< 0.001	1.860	6.424	(1.836; 22.476)	0.004
*CH* + *EH vs. NG*	0.549	1.732	(0.693; 4.325)	0.240	3.188	24.232	(9.438; 62.212)	< 0.001
BMI (Δz-score)	− 0.376	0.687	(0.247; 1.906)	0.471	− 1.391	0.249	(0.083; 0.746)	0.013
Systolic BP (Δz-score)	− 0.231	0.794	(0.527; 1.197)	0.271	− 0.240	0.787	(0.555; 1.116)	0.178
	Model 3: CR vs. NG at the follow-up				Model 4: CH + EH vs. NG at the follow-up			
Intercept	− 1.936	0.144	(0.076; 0.272)	< 0.001	− 2.531	0.080	(0.034; 0.186)	< 0.001
LV geometry at baseline								
*CR vs. NG*	2.307	10.040	(4.356; 23.140)	< 0.001	1.879	6.544	(1.866; 22.952)	0.003
*CH* + *EH vs. NG*	0.536	1.710	(0.683; 4.278)	0.252	3.215	24.892	(9.676; 64.033)	< 0.001
BMI (Δz-score)	− 0.429	0.651	(0.235; 1.805)	0.410	− 1.362	0.256	(0.085; 0.770)	0.015
Diastolic BP (Δz-score)	0.037	1.038	(0.677; 1.590)	0.864	− 0.341	0.711	(0.484; 1.044)	0.082

Δz-score indicates the difference between the baseline value and the follow-up value

*BMI* body mass index, *BP* blood pressure, *CI* confidence interval, *LV* left ventricular, *RRR* relative risk ratio

^*^b, coefficient of the regression model

**Fig. 4:**
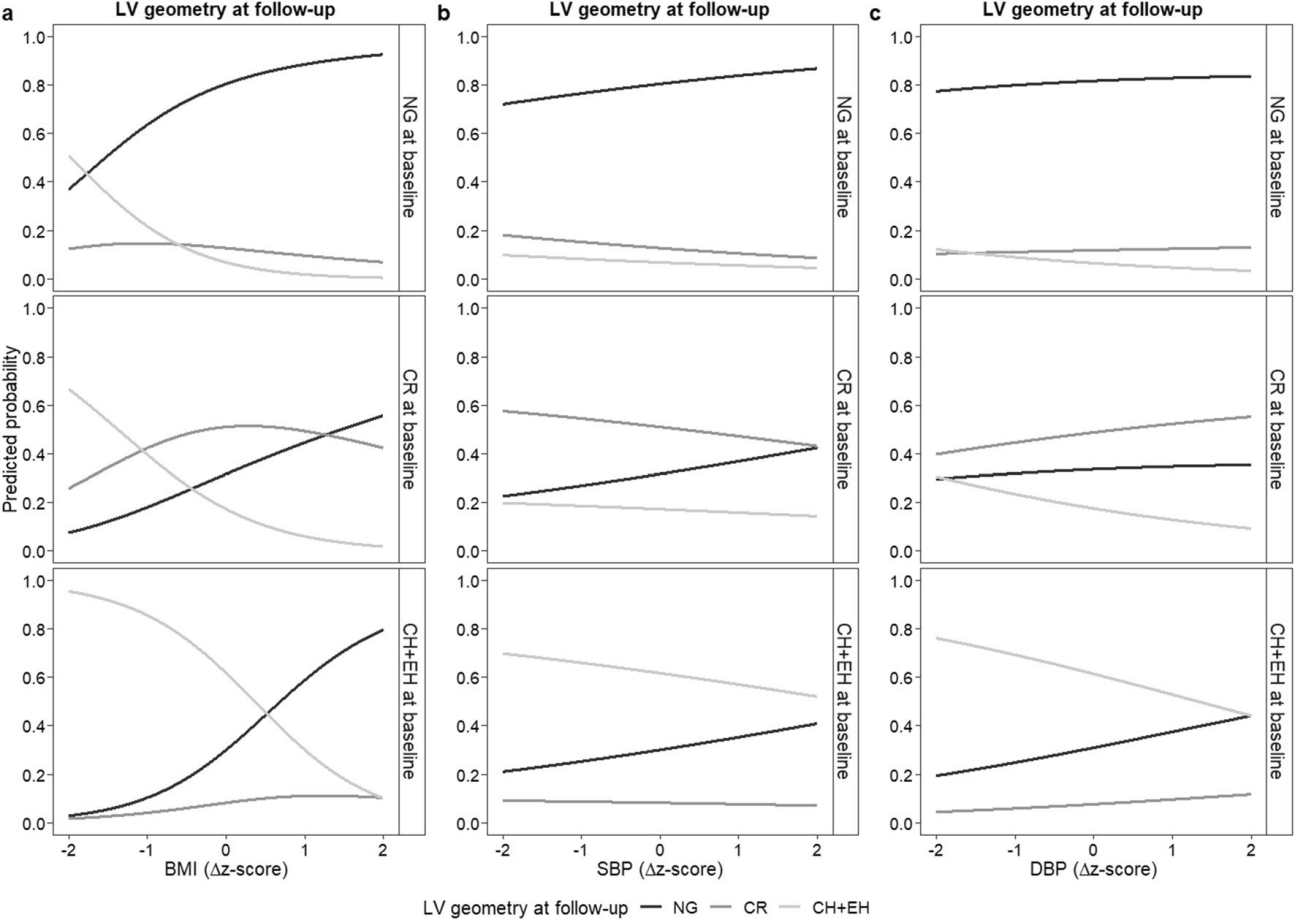
Predicted probabilities of LV geometric pattern (NG, CR, or CH + EH) at follow-up according to the BMI Δz-score (**a**), SBP Δz-score (**b**), or DBP Δz-score (**c**) and LV geometry pattern at baseline (fixed SBP (or DBP) Δz-score = 0 or BMI Δz-score = 0, respectively). *BMI*, body mass index; *CH*, concentric hypertrophy; *CR*, concentric remodeling; *DBP*, diastolic blood pressure; *EH*, eccentric hypertrophy; *LV*, left ventricular; *NG*, normal geometry; *SBP*, systolic blood pressure

In Fig. 4, the predicted probability of being in one of the categories of cardiac geometry at follow-up, stratified by geometry category at baseline, is displayed. At increasing BMI delta z-score (difference between BMI z-score measured at baseline and at follow-up), children with NG at baseline had a higher probability of being NG at follow-up, while the probability of becoming CR remained constant and the probability of becoming CH + EH decreased. In children with CR at baseline, however, at higher BMI delta z-score, the probability of having NG at follow-up increased, whereas that of having CH + EH decreased. Finally, in children with CH + EH at baseline, as delta z-score increased the probability of having NG at follow-up became higher, that of having CR remained constant, whereas the probability of having CH + EH also at follow-up decreased. Predicted probabilities were also linked to SBP and DBP delta z-scores in the same direction as observed for the BMI delta z-scores, but in a less important manner.

## Discussion

Our study shows that correcting unhealthy lifestyles and improving dietary habits in a pediatric population at cardiovascular risk is associated not only with a reduction of both BMI and blood pressure values, but also with a regression of early cardiac organ damage.

The nonpharmacological treatment was accompanied by a significant reduction of the proportion of individuals with pathological conditions (i.e., excess weight or blood pressure > 95^th^ percentile). While all recruited children had at least one pathological condition at baseline (excess weight, high blood pressure, or both), at follow-up 18% of the population showed normal values for both weight and blood pressure and the percentage of hypertensive or obese children was significantly reduced.

It is important to emphasize that these results were obtained without the use of antihypertensive drugs. Some studies have shown that, in children and adolescents with primary or secondary arterial hypertension, normalization of blood pressure values obtained by administration of drug therapy is accompanied by an improvement in echocardiographic parameters [12–15].

In adults, a few studies have examined the efficacy of nonpharmacological approaches to induce LVH regression. It has been shown that dietary sodium restriction is quite effective in lowering LVMI and in simultaneously reducing BP [31] and a recent study performed in hypertensive adults with blood pressure values well controlled by drug treatment showed that the addition of a simple sodium-restricted diet was able to cause a significant reduction in LVMI [32]. However, the Dietary Approaches to Stop Hypertension (DASH) study did not succeed in demonstrating any additional benefit of the DASH diet compared to usual care. An improvement in LVM values was observed only when the DASH diet was administered in conjunction with a weight and exercise controlling program [33]. An observational study highlighted the blood pressure-independent association between moderate weight loss and LVMI reduction in a population of hypertensive patients [34].

To our knowledge, there are no studies in pediatric populations demonstrating a reduction in cardiac mass or improvement in cardiac geometry achieved after lifestyle modification. In a previous study, we showed that dietary-behavioral treatment was able to reduce both blood pressure and BMI z-scores in a population of hypertensive and/or excess weight children and adolescents. At one year of follow-up, the number of individuals who became normotensive and normal weight was 19% and increased to 38% after three years of treatment [17]. Moreover, this approach resulted in an improvement of lipid and glycemic profiles and serum uric acid values [35]. The results of the present study give more information, suggesting that improvement in weight class and in blood pressure category, obtained without the use of drugs, is also associated with a regression of early cardiac organ damage (related to preexisting excess weight and elevated blood pressure values). In our population, the reduction in BMI z-score plays a more important role than the decrease in blood pressure values, both regarding the improvement in cardiac mass values and the reduction of the prevalence of LVH. This finding confirms what has been previously observed in children that the association between LVH and excess weight is stronger than that between LVH and elevated blood pressure values [9, 36]. However, we observed that the reduction of DBP z-score was an independent predictor of reduced prevalence of LVH at follow-up, regardless of whether the child lost weight or not. For each point reduction in BMI z-score, the probability of presenting LVH at follow-up decreased by 80% and for each point reduction in diastolic blood pressure z-score by approximately 40%. This fact suggests that the presence of elevated DBP values constitutes a clinically more serious situation than systolic hypertension alone (frequently encountered in children and adolescents, particularly if overweight or obese) and that normalization of DBP values has a greater impact on early cardiac organ damage than that of SBP values.

Although in our population a positive family history of hypertension was not associated with higher LVMI values and/or a higher prevalence of LVH (data not shown), it is interesting to note that the presence of at least one hypertensive parent was a strongly protective factor with respect to presenting with LVH at follow-up. In children with one or more family members with hypertension, such a possibility was in fact reduced by more than 60%. It is possible to hypothesize that when a family was aware of the clinical implication of being affected by a cardiovascular risk factor such as hypertension, such awareness makes family members more sensitive and compliant in adhering to medical recommendations.

To our knowledge, no data are available describing changes in cardiac geometry associated with nonpharmacological treatment of hypertension and overweight in children. Several authors have demonstrated how overweight, obesity, and hypertension induce pathological changes in cardiac geometry parameters in children, just as observed in adults [8, 37, 38]. Moreover, Guzik et al. recently demonstrated that left ventricular concentric hypertrophy is linked to worse outcomes than eccentric hypertrophy in hypertensive patients, suggesting a role for the type of cardiac remodeling in determining the prognosis in this population [39]. Our study suggests a positive effect of dietary-behavioral treatment on cardiac geometry. This confirms that early cardiac damage in hypertensive and/or overweight/obese children and adolescents could be reversible in a relatively short time, even without the aid of drug therapy. Changes in BMI z-score in our study had an impact of cardiac geometry observed at follow-up, and this impact depended on cardiac geometry at baseline. According to our study results, children with concentric and eccentric hypertrophy at baseline are at greater risk of having hypertrophy also at follow-up and at lower probability of having normal cardiac geometry, as compared to those with normal geometry at baseline. Moreover, our data indicate that children and adolescents with concentric remodeling at baseline are at greater risk of having hypertrophy (concentric or eccentric) and lower possibility of having normal cardiac geometry at follow-up, as compared to those with normal geometry at baseline. These observations underscore the importance of conducting echocardiographic examinations in hypertensive and/or excess weight children and monitoring cardiac ultrasound imaging over time as suggested by several pediatric guidelines [11, 40]. Furthermore, our data suggest that, despite the association of lifestyle modifications with an improvement of altered cardiac geometry and with a reduction of LVH, the presence of concentric remodeling is a factor that negatively affects the possibility of normalization of cardiac geometry, for the same BMI z-score reduction.

Guidelines recommend starting drug therapy immediately in children with hypertension and LVH [10, 11]. The decision to start drug therapy in a child is an important one and needs to be taken very thoughtfully. The definition of LVH in children is based on a statistical criterion and, to our knowledge, there are no studies showing an increase in cardiovascular events in later life in individuals who showed LVH in childhood. Our data suggest that a nonpharmacological approach can yield good results on regression of organ damage. In our opinion, perhaps attempting an intervention based on lifestyle modifications before prescribing medication might also be an approach to consider in hypertensive children with LVH, particularly those who are also excess weight.

It is important to acknowledge a few limitations of this study. Given that in this study it was not possible to include a control group, our results can only be interpreted as showing an association between our lifestyle intervention and echocardiographic changes in the children/adolescents who were followed, without the possibility to establish any cause/effect relationship. A control group was not included because in our opinion (as well as in the opinion of the Ethics Committee) it would have been unethical not to offer any kind of treatment to hypertensive and/or overweight children referred to our center by their family pediatricians. This was an observational study with a sample size depending on the number of assisted children and the availability of the assessments. All patients referred to our center who met the inclusion criteria and gave consent to participate in the study were consecutively recruited for the study. A sample size could not be planned as it was not possible to predict the extent of improvement associated with treatment, this being the first study on this topic. Moreover, we did not obtain records of compliance with the lifestyle interventions, particularly with regard to the low-sodium diet adherence. Indeed, it has been shown that in children a reduction in salt intake causes a decrease in BP and, if continued, can reduce the subsequent increase in BP with age [41]. We have only some indirect evidence of good compliance with the intervention by the fact that most of the children regularly attended all follow-up visits and showed a significant reduction in both body weight and BP; however, we can only talk about an association between positive outcomes and our intervention, not a cause/effect relationship. Finally, because ambulatory blood pressure monitoring (ABPM) was not performed systematically in all children, we could not include this data in our analysis. Therefore, we could not exclude the possibility that some children with high values at office measurement might have normotensive values at ABPM.

In conclusion, this study suggests that in children and teenagers with high blood pressure and/or excess weight, dietary-behavioral treatment may be not only associated with a blood pressure and BMI values at follow-up, but also with a regression of early cardiac involvement, when already present. Our data suggest that an early intervention aimed at correcting erroneous lifestyle and dietary habits can be beneficial starting from childhood and adolescence, especially in individuals with alterations in body weight and blood pressure, indicating that it should be strongly recommended.

More studies on this subject are necessary to confirm a causal relationship between nonpharmacological treatment and cardiac damage regression in hypertensive and/or obese children, and therefore, our results must be confirmed in the future.

### Supplementary information

Below is the link to the electronic supplementary material.

Supplementary file1 (DOCX 155 KB)

Graphical abstract (PPTX 1.86 MB)

## Data Availability

Data are available on reasonable request from the corresponding author.
