# Supplementary material for: Association between lifestyle modifications and improvement of early cardiac damage in children and adolescents with excess weight and/or high blood pressure
Source: Pediatr Nephrol. 2023 Jun 22;38(12):4069–82. doi: 10.1007/s00467-023-06034-5 (PMC10584714; doi:10.1007/s00467-023-06034-5)
Supplement: Supplementary file 1 — Supplementary file1 (DOCX 155 KB) [file 467_2023_6034_MOESM1_ESM.docx]

**Figure S1** Diagram describing the patient selection and follow-up process


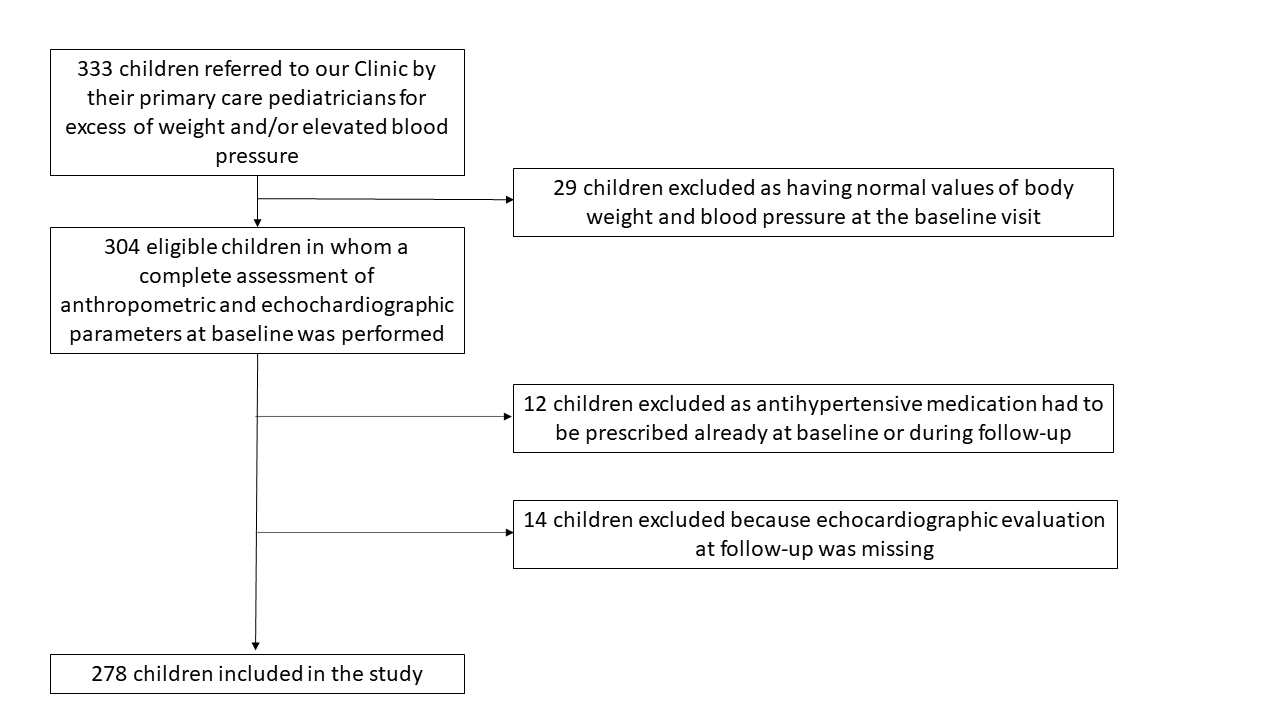


**Table S1.** Sub-groups distribution at baseline and at the follow-up

|  | **Follow-up** | | | |
| --- | --- | --- | --- | --- |
| **Baseline** | **Normal weight with blood pressure <90^th^ percentile**  **(N=49, 17.6%)** | **Normal weight with blood pressure >90^th^ percentile**  **(N=19, 6.8%)** | **Excess weight with blood pressure <90^th^ percentile**  **(N=145, 52.2%)** | **Excess weight with blood pressure >90^th^ percentile**  **(N=65, 23.4%)** |
| **Normal weight with blood pressure <90^th^ percentile**  **(N=0)** | - | - | - | - |
| **Normal weight with blood pressure >90^th^ percentile**  **(N=25, 9.0%)** | 14 (56.0) | 11 (44.0) | 0 (0.0) | 0 (0.0) |
| **Excess weight with blood pressure <90^th^ percentile**  **(N=135, 48.6%)** | 24 (17.8) | 1 (0.7) | 91 (67.4) | 19 (14.1) |
| **Excess weight with blood pressure >90^th^ percentile**  **(N=118, 42.4%)** | 11 (9.3) | 7 (5.9) | 54 (45.8) | 46 (39.0) |

**Table S2.** Effect of gender, age, family history of hypertension, BMI, systolic BP (Model A) or diastolic BP (Model B) and follow-up time on the difference between left ventricular mass index at the follow-up and at baseline in all subjects and separately in children without and with left ventricular hypertrophy at baseline by a multiple linear regression model.

| **All subjects** | | | | | | | |
| --- | --- | --- | --- | --- | --- | --- | --- |
| **Variable** | **Model A** | | |  | **Model B** | | |
|  | b | (95% CI) | P |  | **b** | **(95% CI)** | **P** |
| Intercept | -0.934 | (-4.036; 2.169) | 0.554 |  | -0.905 | (-4.014; 2.203) | 0.567 |
| Gender (males) | 0.384 | (-0.715; 1.484) | 0.492 |  | 0.382 | (-0.718; 1.482) | 0.495 |
| Age (years) | 0.056 | (-0.189; 0.300) | 0.654 |  | 0.065 | (-0.178; 0.308) | 0.600 |
| Family history of hypertension | -0.796 | (-1.957; 0.365) | 0.178 |  | -0.813 | (-1.978; 0.351) | 0.170 |
| BMI (Δz-score) | -4.121 | (-5.694; -2.548) | <0.001 |  | -4.048 | (-5.617; -2.480) | <0.001 |
| Systolic BP (Δz-score) | 0.210 | (-0.396; 0.816) | 0.496 |  | - | - | - |
| Diastolic BP (Δz-score) | - | - | - |  | -0.130 | (-0.779; 0.519) | 0.693 |
| Follow-up time (months) | -0.028 | (-0.086; 0.030) | 0.344 |  | -0.029 | (-0.086; 0.029) | 0.329 |
| **Children without LVH at baseline** | | | | | | | |
| **Variable** | **Model A** | | |  | **Model B** | | |
|  | b | (95% CI) | P |  | **b** | **(95% CI)** | **P** |
| Intercept | 2.985 | (-0.444; 6.414) | 0.088 |  | 2.990 | (-0.432; 6.413) | 0.086 |
| Gender (males) | 0.315 | (-0.958; 1.588) | 0.626 |  | 0.319 | (-0.958; 1.595) | 0.623 |
| Age (years) | -0.198 | (-0.472; 0.075) | 0.155 |  | -0.200 | (-0.471; 0.072) | 0.148 |
| Family history of hypertension | 0.210 | (-1.132; 1.552) | 0.758 |  | 0.211 | (-1.130; 1.551) | 0.757 |
| BMI (Δz-score) | -4.001 | (-5.737; -2.264) | <0.001 |  | -4.005 | (-5.731; -2.279) | <0.001 |
| Systolic BP (Δz-score) | -0.009 | (-0.823; 0.805) | 0.983 |  | - | - | - |
| Diastolic BP (Δz-score) | - | - | - |  | 0.033 | (-0.799; 0.864) | 0.938 |
| Follow-up time (months) | -0.041 | (-0.105; 0.023) | 0.210 |  | -0.041 | (-0.106; 0.023) | 0.208 |
| **Children with LVH at baseline** | | | | | | | |
| **Variable** | **Model A** | | |  | **Model B** | | |
|  | b | (95% CI) | P |  | **b** | **(95% CI)** | **P** |
| Intercept | -7.489 | (-13.206; -1.771) | 0.011 |  | -6.958 | (-12.774; -1.141) | 0.020 |
| Gender (males) | 0.736 | (-1.070; 2.541) | 0.420 |  | 0.776 | (-1.040; 2.592) | 0.398 |
| Age (years) | 0.437 | (-0.005; 0.880) | 0.053 |  | 0.428 | (-0.021; 0.877) | 0.062 |
| Family history of hypertension | -2.643 | (-4.468; -0.818) | 0.005 |  | -2.752 | (-4.592; -0.912) | 0.004 |
| BMI (Δz-score) | -4.028 | (-6.953; -1.102) | 0.007 |  | -3.937 | (-6.874; -1.000) | 0.009 |
| Systolic BP (Δz-score) | 0.411 | (-0.386; 1.207) | 0.309 |  | - | - | - |
| Diastolic BP (Δz-score) | - | - | - |  | -0.189 | (-1.092; 0.715) | 0.679 |
| Follow-up time (months) | 0.017 | (-0.083; 0.117) | 0.739 |  | 0.005 | (-0.095; 0.105) | 0.926 |

b indicates multivariate coefficient; CI, confidence interval; LVH, left ventricular hypertrophy; BMI, body mass index; BP, blood pressure; Δz-score indicates the difference between the baseline value and the follow-up value

**Table S3.** Effect of gender, age, becoming pubescent, family history of hypertension, BMI, systolic BP (Model A) or diastolic BP (Model B) on the difference between left ventricular mass index at the follow-up and at baseline in all subjects and separately in children without and with left ventricular hypertrophy at baseline by a multiple linear regression model.

| **All subjects** | | | | | | | |
| --- | --- | --- | --- | --- | --- | --- | --- |
| **Variable** | **Model A** | | |  | **Model B** | | |
|  | b | (95% CI) | P |  | **b** | **(95% CI)** | **P** |
| Intercept | -0.723 | (-3.846; 2.399) | 0.648 |  | -0.768 | (-3.891; 2.355) | 0.628 |
| Gender (males) | 0.531 | (-0.668; 1.729) | 0.384 |  | 0.527 | (-0.673; 1.728) | 0.388 |
| Age (years) | 0.004 | (-0.262; 0.270) | 0.976 |  | 0.017 | (-0.246; 0.281) | 0.896 |
| Becoming pubescent | -0.324 | (-1.680; 1.032) | 0.638 |  | -0.310 | (-1.668; 1.048) | 0.653 |
| Family history of hypertension | -0.959 | (-2.193; 0.275) | 0.127 |  | -0.962 | (-2.199; 0.274) | 0.127 |
| BMI (Δz-score) | -4.282 | (-6.009; -2.555) | <0.001 |  | -4.194 | (-5.916; -2.472) | <0.001 |
| Systolic BP (Δz-score) | 0.237 | (-0.409; 0.883) | 0.471 |  | - | - | - |
| Diastolic BP (Δz-score) | - | - | - |  | -0.089 | (-0.785; 0.607) | 0.802 |
| **Children without LVH at baseline** | | | | | | | |
| **Variable** | **Model A** | | |  | **Model B** | | |
|  | b | (95% CI) | P |  | **b** | **(95% CI)** | **P** |
| Intercept | 2.322 | (-1.203; 5.847) | 0.195 |  | 2.318 | (-1.195; 5.832) | 0.194 |
| Gender (males) | 0.367 | (-1.035; 1.768) | 0.606 |  | 0.378 | (-1.027; 1.784) | 0.596 |
| Age (years) | -0.222 | (-0.521; 0.077) | 0.144 |  | -0.223 | (-0.519; 0.073) | 0.139 |
| Becoming pubescent | 1.114 | (-0.585; 2.814) | 0.197 |  | 1.121 | (-0.578; 2.821) | 0.194 |
| Family history of hypertension | 0.043 | (-1.385; 1.471) | 0.953 |  | 0.048 | (-1.379; 1.475) | 0.947 |
| BMI (Δz-score) | -3.996 | (-5.933; -2.060) | <0.001 |  | -3.992 | (-5.912; -2.072) | <0.001 |
| Systolic BP (Δz-score) | 0.038 | (-0.837; 0.914) | 0.931 |  | - | - | - |
| Diastolic BP (Δz-score) | - | - | - |  | 0.087 | (-0.814; 0.988) | 0.849 |
| **Children with LVH at baseline** | | | | | | | |
| **Variable** | **Model A** | | |  | **Model B** | | |
|  | b | (95% CI) | P |  | **b** | **(95% CI)** | **P** |
| Intercept | -5.183 | (-10.536; 0.170) | 0.058 |  | -5.208 | (-10.623; 0.206) | 0.059 |
| Gender (males) | 1.091 | (-0.855; 3.037) | 0.268 |  | 1.084 | (-0.872; 3.040) | 0.273 |
| Age (years) | 0.305 | (-0.167; 0.778) | 0.202 |  | 0.322 | (-0.153; 0.797) | 0.182 |
| Becoming pubescent | -1.147 | (-3.080; 0.787) | 0.241 |  | -1.150 | (-3.103; 0.803) | 0.245 |
| Family history of hypertension | -2.906 | (-4.841; -0.971) | 0.004 |  | -2.939 | (-4.891; -0.986) | 0.004 |
| BMI (Δz-score) | -4.806 | (-7.904; -1.707) | 0.003 |  | -4.687 | (-7.797; -1.577) | 0.004 |
| Systolic BP (Δz-score) | 0.373 | (-0.457; 1.202) | 0.374 |  | - | - | - |
| Diastolic BP (Δz-score) | - | - | - |  | -0.004 | (-0.961; 0.952) | 0.993 |

b indicates multivariate coefficient; CI, confidence interval; LVH, left ventricular hypertrophy; BMI, body mass index; BP, blood pressure; Δz-score indicates the difference between the baseline value and the follow-up value

**Table S4.** Effect of left ventricular hypertrophy at baseline, age, gender, family history of hypertension, waist-to-height ratio, systolic BP (Model A) or diastolic BP (Model B) on left ventricular hypertrophy at the follow-up by a multiple logistic regression model

| **Variable** | **Model A** | | |  | **Model B** | | |
| --- | --- | --- | --- | --- | --- | --- | --- |
|  | OR | (95% CI) | P |  | **OR** | **(95% CI)** | **P** |
| LVH at baseline | 16.112 | (7.318; 38.848) | <0.001 |  | 18.020 | (8.006; 44.767) | <0.001 |
| Gender (males) | 1.860 | (0.865; 4.112) | 0.117 |  | 1.897 | (0.872; 4.257) | 0.111 |
| Age (years) | 0.993 | (0.841; 1.172) | 0.934 |  | 0.978 | (0.824; 1.162) | 0.800 |
| Family history of hypertension | 0.262 | (0.102; 0.614) | 0.003 |  | 0.243 | (0.092; 0.580) | 0.002 |
| Waist-to-Height Ratio (Δ%) | 0.923 | (0.845; 1.001) | 0.062 |  | 0.921 | (0.843; 0.998) | 0.054 |
| Systolic BP (Δz-score) | 0.839 | (0.564; 1.224) | 0.369 |  | - | - | - |
| Diastolic BP (Δz-score) | - | - | - |  | 0.620 | (0.397; 0.945) | 0.030 |

OR indicates odds ratio; CI, confidence interval; LVH, left ventricular hypertrophy; BMI, body mass index; BP, blood pressure; Δz-score indicates the difference between the baseline value and the follow-up value.

**Table S5.** Effect of left ventricular hypertrophy at baseline, gender, age, family history of hypertension, BMI, systolic BP (Model A) or diastolic BP (Model B) and follow-up time on left ventricular hypertrophy at the follow-up by a multiple logistic regression model.

| **Variable** | **Model A** | | |  | **Model B** | | |
| --- | --- | --- | --- | --- | --- | --- | --- |
|  | OR | (95% CI) | P |  | **OR** | **(95% CI)** | **P** |
| LVH at baseline | 16.937 | (8.156; 38.262) | <0.001 |  | 17.710 | (8.448; 40.509) | <0.001 |
| Gender (males) | 1.622 | (0.804; 3.342) | 0.181 |  | 1.642 | (0.806; 3.418) | 0.176 |
| Age (years) | 0.946 | (0.800; 1.119) | 0.517 |  | 0.929 | (0.783; 1.101) | 0.396 |
| Family history of hypertension | 0.403 | (0.179; 0.864) | 0.023 |  | 0.367 | (0.159; 0.800) | 0.014 |
| BMI (Δz-score) | 0.229 | (0.073; 0.668) | 0.009 |  | 0.228 | (0.073; 0.672) | 0.009 |
| Systolic BP (Δz-score) | 0.811 | (0.567; 1.140) | 0.235 |  | - | - | - |
| Diastolic BP (Δz-score) | - | - | - |  | 0.644 | (0.430; 0.946) | 0.028 |
| Follow-up time (months) | 1.017 | (0.980; 1.051) | 0.343 |  | 1.015 | (0.979; 1.050) | 0.380 |

OR, odds ratio; CI, confidence interval; LVH, left ventricular hypertrophy; BMI, body mass index; BP, blood pressure; Δz-score indicates the difference between the baseline value and the follow-up value.

**Table S6.** Effect of left ventricular hypertrophy at baseline, age, gender, family history of hypertension, waist-to-height ratio, systolic BP (Model A) or diastolic BP (Model B) and follow-up time on left ventricular hypertrophy at the follow-up by a multiple logistic regression model

| **Variable** | **Model A** | | |  | **Model B** | | |
| --- | --- | --- | --- | --- | --- | --- | --- |
|  | OR | (95% CI) | P |  | **OR** | **(95% CI)** | **P** |
| LVH at baseline | 16.265 | (7.368; 39.376) | <0.001 |  | 18.064 | (8.017; 44.958) | <0.001 |
| Gender (males) | 1.829 | (0.848; 4.054) | 0.128 |  | 1.870 | (0.857; 4.208) | 0.121 |
| Age (years) | 1.002 | (0.846; 1.191) | 0.981 |  | 0.985 | (0.828; 1.175) | 0.865 |
| Family history of hypertension | 0.265 | (0.103; 0.623) | 0.004 |  | 0.245 | (0.093; 0.587) | 0.003 |
| Waist-to-Height Ratio (Δ%) | 0.927 | (0.849; 1.006) | 0.081 |  | 0.924 | (0.845; 1.003) | 0.068 |
| Systolic BP (Δz-score) | 0.839 | (0.565; 1.223) | 0.367 |  | - | - | - |
| Diastolic BP (Δz-score) | - | - | - |  | 0.626 | (0.400; 0.955) | 0.034 |
| Follow-up time (months) | 1.013 | (0.971; 1.050) | 0.515 |  | 1.009 | (0.966; 1.047) | 0.654 |

OR indicates odds ratio; CI, confidence interval; LVH, left ventricular hypertrophy; BP, blood pressure; Δz-score indicates the difference between the baseline value and the follow-up value.

**Table S7.** Effect of left ventricular hypertrophy at baseline, gender, age, becoming pubescent, family history of hypertension, BMI, systolic BP (Model A) or diastolic BP (Model B) on left ventricular hypertrophy at the follow-up by a multiple logistic regression model.

| **Variable** | **Model A** | | |  | **Model B** | | |
| --- | --- | --- | --- | --- | --- | --- | --- |
|  | OR | (95% CI) | P |  | **OR** | **(95% CI)** | **P** |
| LVH at baseline | 22.227 | (9.595; 58.322) | <0.001 |  | 24.916 | (10.493; 67.562) | <0.001 |
| Gender (males) | 2.063 | (0.943; 4.666) | 0.074 |  | 2.098 | (0.944; 4.840) | 0.074 |
| Age (years) | 0.923 | (0.765; 1.112) | 0.401 |  | 0.905 | (0.746; 1.094) | 0.303 |
| Becoming pubescent | 0.891 | (0.383; 2.015) | 0.785 |  | 0.910 | (0.387; 2.078) | 0.824 |
| Family history of hypertension | 0.281 | (0.113; 0.648) | 0.004 |  | 0.242 | (0.093; 0.577) | 0.002 |
| BMI (Δz-score) | 0.224 | (0.062; 0.742) | 0.017 |  | 0.226 | (0.062; 0.766) | 0.020 |
| Systolic BP (Δz-score) | 0.860 | (0.588; 1.237) | 0.421 |  | 0.603 | (0.385; 0.919) | 0.022 |
| Diastolic BP (Δz-score) | - | - | - |  |  |  |  |

OR, odds ratio; CI, confidence interval; LVH, left ventricular hypertrophy; BMI, body mass index; BP, blood pressure; Δz-score indicates the difference between the baseline value and the follow-up value.

**Table S8.** Effect of left ventricular hypertrophy at baseline, gender, age, becoming pubescent, family history of hypertension, waist-to-height ratio, systolic BP (Model A) or diastolic BP (Model B) on left ventricular hypertrophy at the follow-up by a multiple logistic regression model

| **Variable** | **Model A** | | |  | **Model B** | | |
| --- | --- | --- | --- | --- | --- | --- | --- |
|  | OR | (95% CI) | P |  | **OR** | **(95% CI)** | **P** |
| LVH at baseline | 20.116 | (8.574; 53.057) | <0.001 |  | 23.017 | (9.535; 63.066) | <0.001 |
| Gender (males) | 1.797 | (0.805; 4.125) | 0.157 |  | 1.853 | (0.817; 4.346) | 0.145 |
| Age (years) | 0.972 | (0.815; 1.161) | 0.749 |  | 0.954 | (0.795; 1.145) | 0.611 |
| Becoming pubescent | 0.976 | (0.408; 2.272) | 0.956 |  | 1.025 | (0.425; 2.412) | 0.955 |
| Family history of hypertension | 0.222 | (0.082; 0.544) | 0.002 |  | 0.201 | (0.072; 0.505) | 0.001 |
| Waist-to-Height Ratio (Δ%) | 0.914 | (0.831; 0.995) | 0.047 |  | 0.912 | (0.830; 0.993) | 0.042 |
| Systolic BP (Δz-score) | 0.874 | (0.582; 1.293) | 0.504 |  | - | - | - |
| Diastolic BP (Δz-score) | - | - | - |  | 0.607 | (0.380; 0.940) | 0.029 |

OR indicates odds ratio; CI, confidence interval; LVH, left ventricular hypertrophy; BP, blood pressure; Δz-score indicates the difference between the baseline value and the follow-up value.

**Table S9**. Left ventricular geometry distribution at baseline and follow-up

|  | **Follow-up** | | | |
| --- | --- | --- | --- | --- |
| **Baseline** | **NG**  **(N=170, 61.2%)** | **CR**  **(N=46, 16.5%)** | **CH**  **(N=24, 8.6%)** | **EH**  **(N=38, 13.7%)** |
| **NG (N=130, 46.8%)** | 110 (84.6) | 14 (10.8) | 1 (0.8) | 5 (3.8) |
| **CR (N=47, 16.9%)** | 18 (38.3) | 23 (48.9) | 5 (10.6) | 1 (2.1) |
| **CH (N=51, 18.3%)** | 15 (29.4) | 8 (15.7) | 15 (29.4) | 13 (25.5) |
| **EH (N=50, 18.0%)** | 27 (54.0) | 1 (2.0) | 3 (6.0) | 19 (38.0) |

NG, normal geometry; CR, concentric remodeling; CH, concentric hypertrophy; EH, eccentric hypertrophy
