# Supplementary material for: Association between lifestyle modifications and improvement of early cardiac damage in children and adolescents with excess weight and/or high blood pressure
Source: Pediatr Nephrol. 2023 Jun 22;38(12):4069–82. doi: 10.1007/s00467-023-06034-5 (PMC10584714; doi:10.1007/s00467-023-06034-5)
Supplement: Supplementary file 2 — Graphical abstract (PPTX 1.86 MB) [file 467_2023_6034_MOESM2_ESM.pptx]

## Slide 1
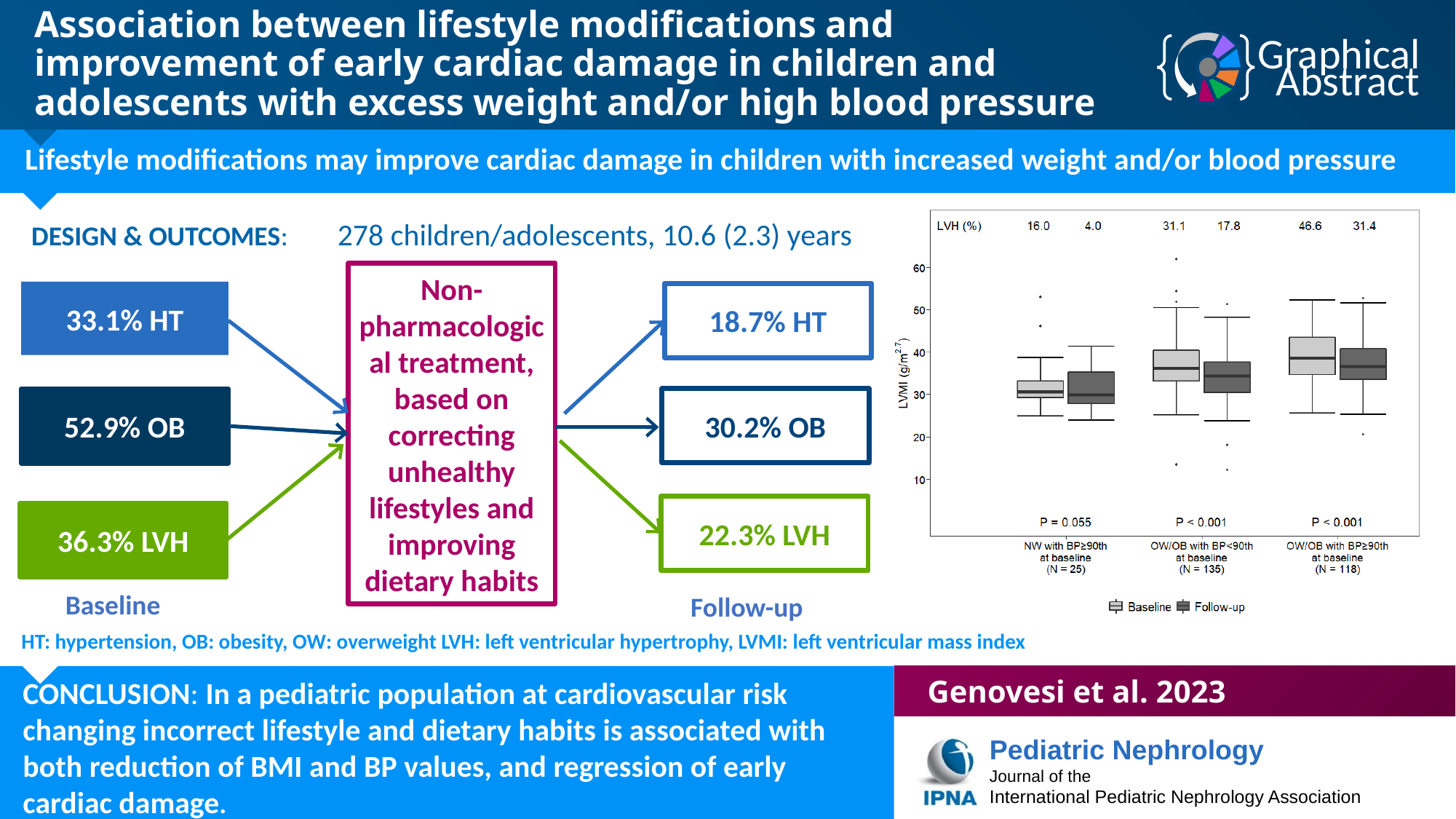

Association between lifestyle modifications and improvement of early cardiac damage in children and adolescents with excess weight and/or high blood pressure
Lifestyle modifications may improve cardiac damage in children with increased weight and/or blood pressure
DESIGN & OUTCOMES: 278 children/adolescents, 10.6 (2.3) years
Non-pharmacological treatment, based on correcting unhealthy lifestyles and improving dietary habits
33.1% HT
18.7% HT
30.2% OB
52.9% OB
22.3% LVH
36.3% LVH
Baseline
Follow-up
HT: hypertension, OB: obesity, OW: overweight LVH: left ventricular hypertrophy, LVMI: left ventricular mass index
Genovesi et al. 2023
CONCLUSION: In a pediatric population at cardiovascular risk changing incorrect lifestyle and dietary habits is associated with both reduction of BMI and BP values, and regression of early cardiac damage.
